# Visual outcome after fractionated stereotactic radiation therapy of benign anterior skull base tumors

**DOI:** 10.1007/s11060-014-1399-0

**Published:** 2014-02-15

**Authors:** Arnar Astradsson, Anne Katrine Wiencke, Per Munck af Rosenschold, Svend-Aage Engelholm, Lars Ohlhues, Henrik Roed, Marianne Juhler

**Affiliations:** 1 Department of Neurosurgery, Rigshospitalet, Copenhagen, Denmark; 2 Department of Ophthalmology, Rigshospitalet, Copenhagen, Denmark; 3 Department of Ophthalmology, Glostrup Hospital, Copenhagen, Denmark; 4 Department of Radiation Oncology, Finsen Center, Rigshospitalet, Copenhagen, Denmark

**Keywords:** Fractionated stereotactic radiation therapy, Visual outcome, Radiation induced optic neuropathy, Tumor control, Anterior skull base meningiomas, Pituitary adenomas

## Abstract

To determine visual outcome including the occurrence of radiation induced optic neuropathy (RION) as well as tumor control after fractionated stereotactic radiation therapy (FSRT) of benign anterior skull base meningiomas or pituitary adenomas. Thirty-nine patients treated with FSRT for anterior skull base meningiomas and 55 patients treated with FSRT for pituitary adenomas between January 1999 and December 2009 with at least 2 years follow-up were included. Patients were followed up prospectively with magnetic resonance imaging scans, visual acuity and visual field examinations. RION was found in four (10 %) patients with anterior skull base meningiomas and seven patients (13 %) with pituitary adenomas. The five-year actuarial freedom from 25 % RION visual field loss was 94 % following FSRT. Actuarial 2-, 5- and 10-year tumor control rates were 100, 88.4 and 64.5 % for anterior skull base meningiomas and 100, 98.2 and 94.9 % for pituitary adenomas, respectively. Patients with an impaired visual field function pre-FSRT were more likely to experience worsened function (*p* = 0.016). We found that RION, was a relatively uncommon event, in a large prospective cohort of patients that were systematically monitored following FSRT of benign anterior skull base tumors. Long term tumor control was favorable, especially for pituitary adenomas.

## Introduction

Stereotactic radiosurgery (SRS) and fractionated stereotactic radiotherapy (FSRT) are image-guided highly precise irradiation therapies used for tumor tissue or malformations, with the aim of maximizing the irradiation dose to the target and minimizing the dose to nearby normal tissue structures [1]. SRS/FSRT is the treatment of choice if open surgery is considered too risky; as an adjuvant treatment to surgery; when complete surgical resection is not possible or with residual or recurrent disease [2]. Also, in the case of hormone secreting pituitary tumors, FSRT may be indicated to treat hypersecretion [1]. Accordingly, the most frequent indications for FSRT or SRS in the anterior skull base are meningiomas of the cavernous sinus and pituitary tumors with parasellar extension due to risk of cranial nerve injury and non-radicality with surgery for such lesions [3]. When choosing this treatment modality for lesions close to eloquent brain structures, it may be feasible to choose the fractionated therapy (FSRT), which combines the high accuracy of stereotactic radiation with the biological advantage of fractionation [1, 2]. Few long-term reports on visual complications after FSRT for anterior skull base tumors exist [4]. Furthermore, studies correlating irradiation dose to the optic pathways are few. We here describe visual outcome and the occurrence of radiation induced optic neuropathy (RION) and tumor control after FSRT in a prospective cohort of benign anterior skull base meningiomas and pituitary adenomas.

## Methods

### Study protocol

Patients were recruited consecutively and prospectively into a local treatment protocol and database for anterior skull base meningiomas and pituitary adenomas during the period January 1999–December, 2009 at Rigshospitalet, Copenhagen, Denmark. This report includes patients 18 years of age and older at the time of treatment. Diagnosis was based on histological confirmation, or typical radiographic appearance for cases without surgery. Only patients with ophthalmological examinations and magnetic resonance imaging (MRI) scans at least 2 years after FSRT treatment were included in the analysis. Patients with malignant tumors were excluded.

### Patient population

A total of 44 patients with anterior skull base meningiomas and 66 patients with pituitary adenomas were treated with FSRT. One patient diagnosed with a malignant meningioma was excluded. Of the remaining 43 patients with anterior skull base meningiomas, 2 patients died of unrelated causes within 2 years of treatment, 1 patient emigrated and further 1 patient was lost to follow-up, leaving 39 patients with ≥2 years follow-up available for analysis. Of the 66 patients with pituitary adenomas, 2 patients died of unrelated causes within 2 years of treatment, 3 patients emigrate, and further 6 patients were lost to follow-up, leaving 55 patients with ≥2 years follow-up available for analysis. For age and sex distribution, tumor characteristics, including tumor volume and details of surgical treatment, see Table 1.

**Table 1:** Patients, tumor characteristics and surgical treatment

Anterior skull base meningiomas	
Total 39	
Females	30 (77 %)
Males	9 (23 %)
Mean age (years)	56 (37–78)
Pituitary adenomas	
Total	55
Females	29 (53 %)
Males	26 (47 %)
Mean age (years)	48 (18–73)
Anterior skull base meningiomas, tumor location	
Cavernous sinus	23 (59 %)
Clival/Petroclival	12 (31 %)
Sphenoid wing	3 (8 %)
Optic canal	1 (2 %)
Anterior skull base meningiomas, tumor volume	21.02 cc (mean, range 0.33–152.50)
Pituitary adenomas, tumor extension	
Intrasellar	5 (9 %)
Extrasellar	50 (91 %)
Pituitary adenoms, tumor volume	6.66 cc (mean, range 0.00–41.83)
Pituitary adenomas, secretory status	
Somatotrophic (GH secreting)	34 (62 %)
Corticotrophic (ACTH secreting)	4 (7 %)
Lactotrophic (prolactin secreting)	2 (4 %)
Null adenoma (non-secreting)	15 (27 %)
Operation prior to FSRT	
Anterior skull base meningiomas	
Total	25 (64 %)
One/two/three operations	13/8/4
Median time from last operation until FSRT:	28 months (range 1–102)
Pituitary adenomas	
Total	49 (91 %)
One/two/three/four operations	24/16/9/1
Median time from last operation until FSRT:	10 months (range 1–134)

### Fractionated stereotactic radiation therapy

In all cases, a linear accelerator was used to deliver the stereotactic irradiation treatments; a Clinac 600SR (Varian Medical Systems) machine was used 1999-2008, and was thereafter replaced by 3 NovalisTx^©^ machines (Varian Medical Systems, Palo Alto, CA, and BrainLab, München, Germany). Micro-Multi-leaf collimators were used since 2002, and fixed cones for the first patients. Initially, we used circular collimators, 0.5–3.5 cm diameter with 0.5 cm increments between sizes. From August 2000 and onwards, collimation and field shaping has been provided by micro-multileaf collimators after upgrading. The treatment planning was performed using a system dedicated for stereotactic radiotherapy (XKnife, RSA, US, and BrainScan and iPlan, BrainLab, Munich, Germany). A pencil beam-based dose calculation algorithm was used for all patients from 2001 to 2002, earlier dosimetry were based on Clarkson integration. All cases were treated with multiple FSRT. An individual mask of the head was made during the planning process and was used during all the treatments. The mask was fixed to the board during the radiation, providing immobilization. A fusion of MR and computed tomography (CT) scans was used as a visual treatment plan, whereby the gross target volume (GTV) was estimated. The target volume was defined and treated without setup margins. The prescription dose of 54 Gy was typically prescribed to the 90 % isodose contour, and the 90 % isodose contour was encompassing the target volume. Prescribed tumor radiation doses were 1.8 Gy per dose given in 30 fractions or 2.0 Gy per dose given in 27 fractions (see also Table 2). Nineteen patients received treatment with intensity modified radiation therapy (IMRT) and 75 patients received dynamic three-dimensional Conformal Radiation Therapy (3DCRT arcs).

**Table 2:** Prescribed tumor treatment doses to the 90 % isodose line, combined optic apparatus (COP) radiation doses (median of maximum doses) and proximity to the anterior visual pathways

Anterior skull base meningiomas	
Tumor dose 1.8 × 30	31 (79 %)
Tumor dose 2.0 × 27	8 (21 %)
COS (optic nerves + chiasm + tracts)	53.3 (range 6.6–63.6)
Tumor-anterior visual pathways direct contact	24 (61.5 %)
Pituitary adenomas	
Tumor dose 1.8 × 30	35 (64 %)
Tumor dose 2.0 × 27	20 (36 %)
COS (optic nerves + chiasm + tracts)	54.6 (range 17.4–64.2)
Tumor-anterior visual pathways direct contact	12 (22 %)

### Visual pathways, definition and follow-up

Prospective ophthalmological follow-up after FSRT was scheduled at 9 months, 2 years, 3.5 years, 5 years, 7 years and 10 years after treatment. Visual acuity was measured with a Snellen eye chart and an improvement in visual acuity was defined as a gain of two or more Snellen lines, and worsening was defined as a loss of two or more lines. Visual fields were quantified with Campimetry, Goldmann dynamic perimetry and Octopus static perimetry. The central 30° of the visual fields for each eye separately, were subdivided into an inner, middle and outer circle area, and each circle subdivided into 8 fields, resulting in a maximum of 24 defects per eye. An improvement in visual fields was defined as a gain of 1/24 or more and worsening was defined as a loss of 1/24 or more of the fields for any eye.

### Optic radiation dose and statistical analysis

The combined optic structure (COS) (optic nerves, chiasm and tracts) was outlined by BrainScan or BrainLab autocontouring function. The presence or absence of direct contact between tumor and anterior visual pathways was determined from the 3-dimensional dose plan MRI scans. Dosimetry data were extracted from the dose plan records. Most patients had only the COS delineated. A few patients had data separately for optic chiasm, tracts and nerves on the left and right sides (20 out of 94). For RION events analysis, dose-data were corrected to a radiobiologically equivalent dose in 2 Gy per fraction using the linear-quadratic model and an alpha/beta ratio of 3, which was used subsequently in the analysis. Further, only the maximum dose of the COS was considered, extracting the maximum of the dose delivered to the chiasm, optic nerves and optic tracts.

The visual outcome was categorized as RION visual acuity event in case acuity dropped from greater than 0.2–0.2 or less on one or both eyes. Visual field decrease by 4.2, 12.5, 25 and 50 % or more (1/24, 3/24, 6/24 and 12/24 fields) on one or both eyes, respectively was categorized as an RION visual field event. Patients with tumor growth or the occurrence of other optic lesion following FSRT were censored. We investigated if both RION field and acuity events were associated with maximum radiation dose to the COS, dose per fraction, number of operations performed pre-FSRT, age at FSRT, diagnosis and tumor size at baseline using Cox regression models. A *p* value of 0.004 was considered significant (Bonferroni correction 0.05/14).

In a post hoc analysis we investigated the association of maximum radiation dose to COS, treatment technique (IMRT or 3DCRT), tumor size and if the tumor was in direct contact with the optic nerves/chiasm (SPSS, IBM, US). Visual acuity and field function at baseline, categorized as “intact” or “impaired”, was compared to the function following FSRT categorized as “improved”, “unchanged” or “worsened”. Patients with an “intact” status at baseline were by definition not considered able to “improve”. The risk of having worsened function was compared for the groups where function was intact and impaired at baseline (Fisher’s exact test, two-tailed) using the R statistical package (R version 3.0.1, 2013-05-16). Also, the difference of visual acuity and fields function at baseline for meningioma and pituitary adenoma patients were compared (Fisher’s exact test, two-tailed). In the post hoc analysis, a *p*-value of 0.05 was considered significant.

### Tumor control measures

Prospective neuroimaging follow-up after FSRT was scheduled at 9 months, 2 years, 3.5 years, 5 years, 7 years and 10 years after treatment, with T1-weighted MRI images of the head, after application of Gadolinium, in the three planes in all studies. Tumor size was measured in three dimensions: antero-posterior, lateral and cranio-caudal. Largest tumor dimensions were defined on pre-treatment and the latest post-treatment contrast MRI studies. Tumor control was defined as regression or stable tumor size. Only a change of 2 mm or more in any dimension was considered a change in tumor size. Tumor volume in cm^3^ (cc) was defined using treatment planning software.

## Results

### Visual outcome

Median follow-up after FSRT for anterior skull base meningiomas was 5.4 years (range 2.7–10.6) and for pituitary adenomas 6.8 years (range 3.0–13.4). Visual acuity for anterior skull base meningiomas of the left eye was 0.86 (±0.36) pre- and 0.84 (±0.36) post-FSRT and of the right eye 0.77 (±0.39) pre- and 0.76 (±0.41) post-FSRT. Visual acuity for pituitary adenomas of the left eye was 0.95 (±0.24) pre- and 0.95 (±0.28) post-FSRT and of the right eye 0.85 (±0.30) pre- and 0.87 (±0.33) post-FSRT. Visual baseline and outcome data are presented in Table 3. Baseline visual fields were significantly better for the pituitary adenoma group (*p* = 0.049), while visual acuity was not significantly different at baseline (*p* = 0.291) for meningioma and pituitary adenoma patients. Meningioma patients with impaired visual fields at baseline had a slightly higher risk for worsened function following FSRT (*p* = 0.068). Pituitary adenoma patients with impaired visual acuity at baseline had a slightly higher risk for worsened function following FSRT (*p* = 0.064). Pooling the data for both diseases, the risk of worsened visual fields and visual acuity following FSRT, was elevated for patients with an impaired function at baseline (*p* = 0.016, 0.067, respectively).

**Table 3:** Visual function

	Baseline status	Improved	Unchanged	Worsened	Fisher’s exact test
Anterior skull base meningiomas	Visual acuity intact	–	18	1	*p* = 1.000
	Visual acuity impaired	2	16	2	
	Visual fields intact	–	21	1	*p* = 0.068
	Visual fields impaired	3	9	5	
Pituitary adenomas	Visual acuity intact	–	33	1	*p* = 0.064
	Visual acuity impaired	2	15	4	
	Visual fields intact	–	37	5	*p* = 0.192
	Visual fields impaired	3	6	4	

Fisher’s exact test (two tailed). Comparing the fraction of patients ending up with an unchanged or improved versus worsened function, on last follow-up, who had an intact versus impaired visual function at baseline

### Radiation induced optic neuropathy (RION)s

Eighty-eight patients were evaluable with respect to RION after excluding six patients with pre-treatment blindness due to tumor compression (two patients), cataract, keratitis, optic neuritis and bilateral glaucoma. Radiation-induced visual symptoms were identified in 15 of the 94 patients in the whole series, including 11 patients with RION and 4 patients with lens or cornea damage. Of the 39 patients with anterior skull base meningiomas, 4 patients (10.3 %) developed new visual field defects after FSRT, at 6.1–25.5 months after FSRT, in the absence of other optic lesions or tumor growth, and were therefore considered to have RION. Of the 55 patients with pituitary adenomas, 7 patients (12.7 %) developed new visual field defects, and one of these also developed visual acuity loss, at 9.0–79.6 months after FSRT, in the absence of other optic lesions or tumor growth, and were therefore considered to have RION.

### Optic radiation dose, tumor-optic direct contact and statistical analysis

Fourteen, 21 and 6 patients received a COS dose of 55–60, 60–65 and 65–68 Gy (2-Gy equivalent dose), respectively (see Table 2 for details). With only one RION acuity loss event observed, no meaningful statistical analysis could be performed for RION acuity loss. For the patient with RION acuity event, the maximum dose to COS was a modest 29 Gy. RION field loss of 1–2, 3–5, 6–11 and 12–24 fields out of 24 were observed for three, five, six and four eyes, respectively, out of the evaluable cohort.

**Fig. 1:**
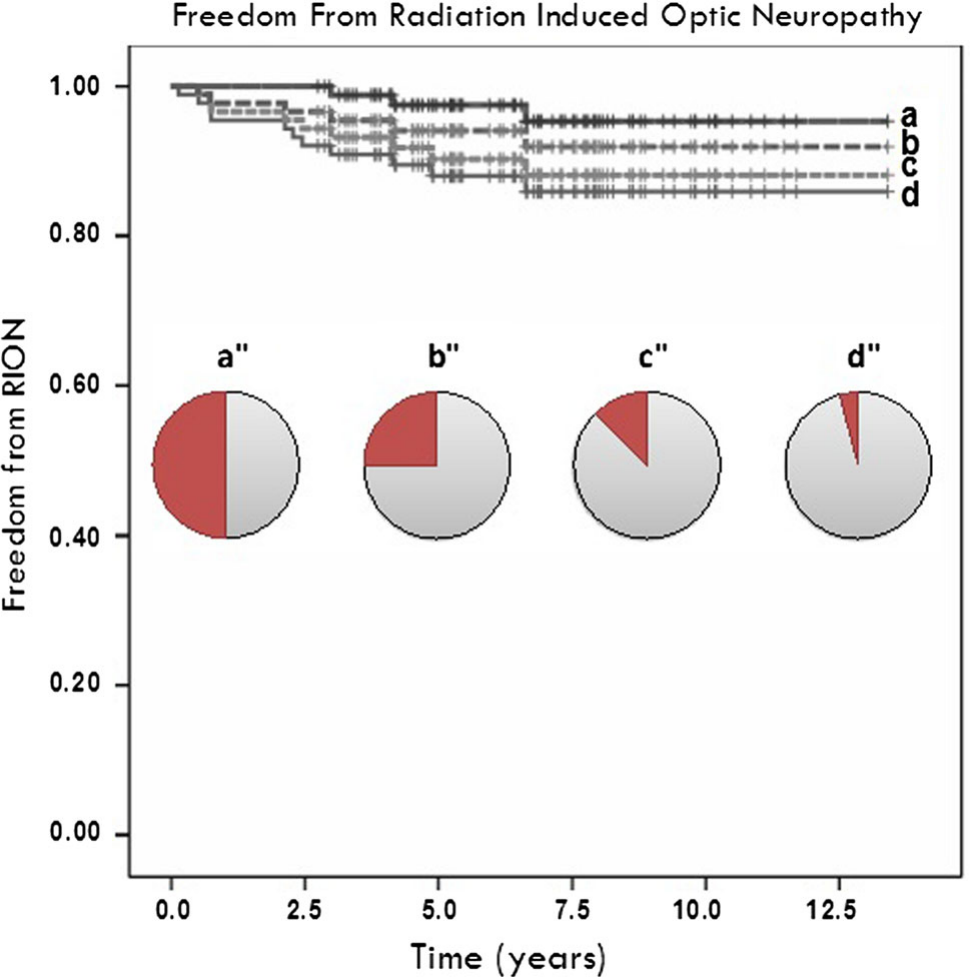
Actuarial data for RION field loss for all patients, with patients having **a** loss of half or more, **b** a quarter or more, **c** 1/8th or more and **d** 1/24th or more of the visual field for one or both eyes

Tumor was found to be in direct contact with the anterior visual pathways in 24 (61.5 %) cases of anterior skull base meningiomas and in 12 (20 %) cases of pituitary adenomas, with a variable degree of compression of the optic nerves or chiasm (Table 2). The association between tumor volume (cc), maximum COS dose (Gy), tumor-optic direct contact (0 = no, 1 = yes), IMRT use (0 = no, 1 = yes) and disease (pituitary = 0, meningioma = 1) was investigated using Spearman signed rank test. COS maximum dose, was associated with tumor-optic direct contact (ρ = 0.312, *p* = 0.002). Also, IMRT use resulted in lower doses to the COS (ρ = −0.415, *p* < 0.001). Larger tumors indicated increased dose to the COS (ρ = 0.236, *p* = 0.022) and were more likely to be associated with compression or distortion of the optical structures (ρ = 0.434, *p* < 0.001). However, maximum dose to COS and tumor size at FSRT were non-significantly associated with RION field loss events. Also, age, diagnosis, dose per fraction (2.0 or 1.8 Gy) and number of operations before radiation therapy were all non-significantly associated with RION field loss events. Actuarial data for RION field loss are shown in Fig. 1; 5-year Kaplan–Meier estimated freedom from 25 % RION visual field loss was 94 %.

### Long-term tumor control after FSRT

**Fig. 2:**
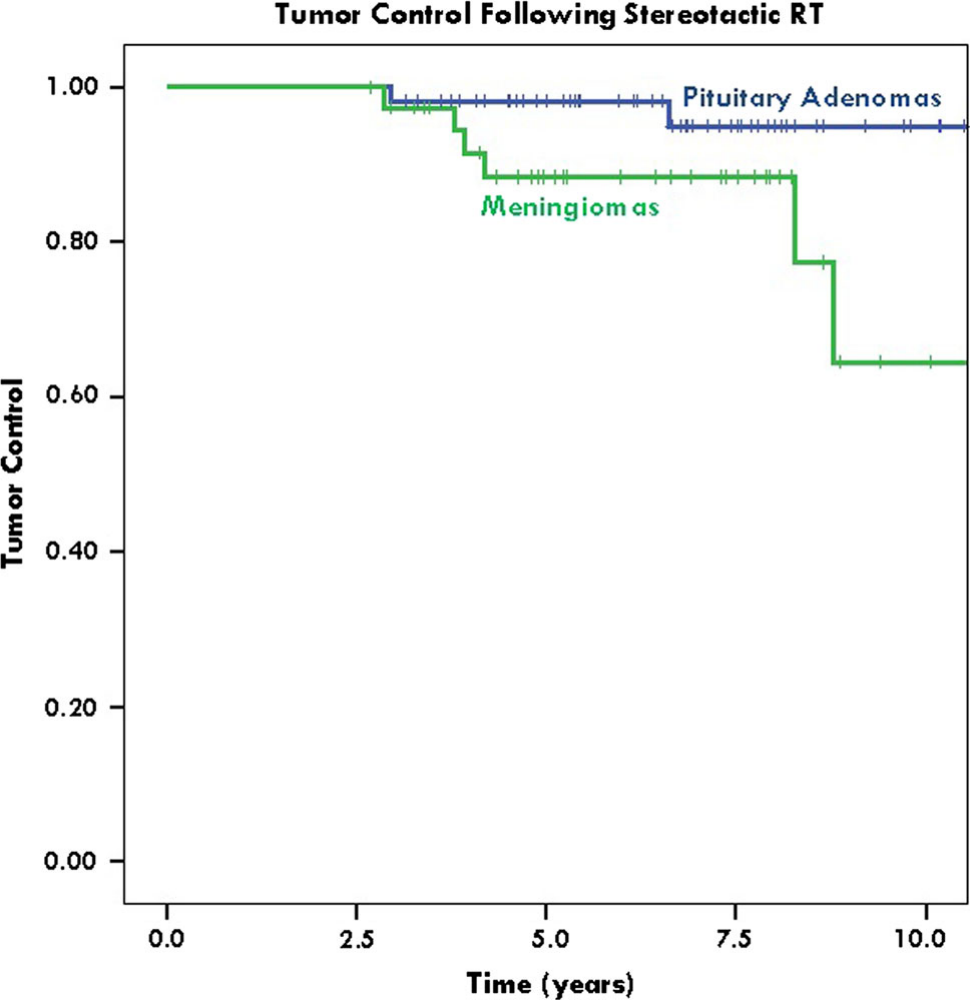
Kaplan–Meier plots of long term tumor control, with 95 % confidence interval lines. Actuarial 2-, 5- and 10-year tumor control rates post FSRT were 100, 88.4 % (95 % CI 72.2-94.9) and 64.5 % (95 % CI 28.3–85.9), respectively, for anterior skull base meningiomas, and 100, 98.2 % (95 % CI 87.5-98.5) and 94.9 % (95 % CI 79.8-98.5), respectively, for pituitary adenomas

For anterior skull base meningiomas, median follow-up was 6.4 years (range 2.7–11.7). Actuarial 2-, 5- and 10-year tumor control rates post FSRT were 100, 88.4 (95 % CI 72.2–94.9) and 64.5 % (95 % CI 28.3–85.9), respectively (Fig. 2). Mean tumor volume pre-FSRT was 21.02 cc (range 0.33–152.50) and post-FSRT 22.62 cc (range 0.16–184.70). One patient (2.6 %) underwent reoperation 55 months after FSRT due to symptomatic tumor progression causing exophthalmos.

For pituitary adenomas, median follow-up was 6.8 years (range 2.9–13.4 years). Actuarial 2-, 5- and 10-year tumor control post FSRT were 100, 98.2 % (95 % CI 87.5–98.5) and 94.9 % (95 % CI 79.8–98.5), respectively (Fig. 2). Mean tumor volume pre-FSRT was 6.66 cc (range 0.00–41.83) and post FSRT 3.75 cc (range 0.00–38.13). One patient (2.0 %) underwent reoperation 35 months after FSRT due to symptomatic tumor progression causing visual deterioration.

## Discussion

This is, to our knowledge, the largest prospective study of the occurrence of RION after linear accelerator-based FSRT of anterior skull base meningiomas and pituitary adenomas. We found the occurrence of radiation-induced optic neuropathy after FSRT to be as high as 10 % in patients with anterior skull base meningiomas and 13 % with pituitary adenomas, although in the majority of most cases these were mild to moderate. Only one patient displayed marked visual acuity loss and worsening of visual fields was mild to moderate in most cases. Importantly, vision remained stable in a majority of patients and improved in a subset of patients.

Our data show a lower occurrence of RION than reported by Emami [5], and support the view presented in the recent review by the QUANTEC initiative, reporting expected risks of 3–7 and 7–20 % of RION in the dose range 55–60 and above 60 Gy, respectively, for maximum dose to the optic nerves [4]. A comparison with historical risk data is often complicated, though, and differences may be due to treatment planning, delivery aspects and clinical evaluations and other unknown factors.

In comparison to RION acuity loss, RION field loss was more prevalent, but appeared not to be associated with the maximum dose to the optic structures. Furthermore, although the radiation dose to the optic structures was higher for larger tumors and tumors compressing the optic structures, this increased dose was not associated with a difference in the rate of RION events. There might be several reasons for this lack of association. For example, MRI-tractography could be used for improved definition the optic pathways, but was not available for the present analysis. Also, minor positioning inaccuracies can cause rather substantial change in the maximum radiation dose due to the sharp dose-gradients of the treatment plans. Additionally, the dose-volume relationships are complex. A more in-depth analysis addressing these points is however beyond the scope of this work. SRS and FSRT both aim at sparing nearby eloquent structures, such as the optic chiasm [1]. Fractionated therapy has the biological advantage over single dose radiosurgery that it divides the total radiation dose into several smaller doses, so that a high radiation dose to the healthy structures in one single session is avoided [1]. In particular, SRS may not be suitable for lesions in close proximity to the visual pathways, due to a dose-limiting factor, although SRS studies have not been unequivocal, in particular regarding the dose–response tolerance of the optic pathways. For example, it was found that <10 Gy to lesions of the cavernous sinus in a single dose did not cause risk to the optic pathways, while 10–15 and >15 Gy caused a 27 and 78 % incidence of optic neuropathy, respectively [6]. Other SRS studies of perioptic tumors have reported variable results. Two large Gamma Knife (GK)-SRS studies reported a 0 and 1.9 % incidence of RION, respectively, at optic doses of 10 Gy [7, 8], but another study reported a 4 % incidence at an optic dose of 3, 1 Gy [9]. Large LINAC-SRS studies have reported a low incidence of RION of 0–2.8 % at optic doses less than 10 Gy [10–12]. Finally, a large combined LINAC/GK-SRS study found a 24 % risk of RION at optic doses greater than 8 Gy, but none at doses less than 8 Gy [13].

Our visual outcome data compare somewhat unfavorably with earlier series of tumors of the anterior skull base region treated with FSRT, with several studies reporting a low 0–6 % incidence of visual loss for meningiomas around the anterior visual pathways [3, 14–20] and similarly for pituitary adenomas, in the range 0–9 % [2, 21–29]. However, direct comparison with these previous studies is difficult, since median follow-up for most of them was significantly shorter [2, 3, 15, 16, 19, 20, 22, 24–27, 29] and several included a number of patients with less than 2 years follow up [2, 16, 20, 22, 24, 26, 27, 29, 30]. Indeed in our series, RION was in many cases a late event. More importantly, formal ophthalmological examinations were only consistently available in approximately half of the previous series [2, 17, 18, 20, 22–24, 26, 27, 31] and only a minority of the studies reported optic radiation dose [2, 17, 18, 23–25].

More recently, Stiebel-Kalish and colleagues [32] reported 16 patients with meningiomas around the anterior visual pathways, treated with LINAC-FSRT, with regular ophthalmological examinations, and in line with our outcome data, found a 12 % incidence of overall worsening of visual function. However, Kocher and colleagues, in another recent LINAC-FSRT series of 29 patients with perioptic tumors with a maximal total dose of 53.3 an 54.3 Gy to the optic nerves and chiasm, respectively, and maximal fractions of 1.86, reported only two new cases of disease related visual loss, but patients with short follow up were included [33].

In our study, tumor was found to be in direct contact with the anterior visual pathways in 24 (61.5 %) cases of anterior skull base meningiomas and in 12 (20 %) cases of pituitary adenomas and the optic apparatus as is often the case with these tumors, making it inevitable with current techniques that the optic apparatus often receives irradiation as high as the tumor itself. Similarly, Adler et al. [34] in a Cyberknife multisession radiosurgery series of perioptic tumors, all tumors were located within 2 mm of the optic apparatus, although they reported a high visual preservation of 94 % of cases.

The 5 year tumor control rate of 88.4 % for the anterior skull base meningiomas did not differ significantly from that of other FSRT series reporting a tumor control rate of 88–98 % [14, 16, 30, 32, 35, 36] and the 5 year tumor control rate for the pituitary adenomas of 98.2 %, compared favorably with the rates of 93–99 % reported in other FSRT series [21–24, 27]. Furthermore, although tumors close to the visual pathways treated with FSRT tend to be larger, our 5 syear tumor control rates seem to be comparable even with those reported for large LINAC-SRS [10–12, 37] or GK-SRS series [7, 38, 39].

In conclusion, balanced against the risks of uncontrolled tumor growth it may be concluded that FSRT is a relatively safe and effective treatment of these tumors. Improved dose planning techniques may be able to reduce the incidence of visual complications further.
